# Catecholaminergic and Cholinergic Systems Mediate Beneficial Effect of Vortioxetine on Diabetes-Induced Neuropathic Pain

**DOI:** 10.3390/biomedicines11041137

**Published:** 2023-04-10

**Authors:** Nazlı Turan Yücel, Ümmühan Kandemir, Umut İrfan Üçel, Ümide Demir Özkay, Özgür Devrim Can

**Affiliations:** 1 Department of Pharmacology, Faculty of Pharmacy, Anadolu University, 26470 Eskişehir, Turkey; udemir@anadolu.edu.tr (Ü.D.Ö.); ozgurdt@anadolu.edu.tr (Ö.D.C.); 2 Vocational School of Health Services, Bilecik Şeyh Edebali University, 11230 Bilecik, Turkey; ummuhan.kandemir@bilecik.edu.tr; 3 Vocational School of Health Services, Bayburt University, 69000 Bayburt, Turkey; umutucel@bayburt.edu.tr

**Keywords:** allodynia, diabetes, dynamic plantar test, hyperalgesia, Randall–Selitto test, vortioxetine

## Abstract

The therapeutic potential of vortioxetine on mechanical hyperalgesia/allodynia was investigated in rats with streptozotocin-induced diabetes, and its possible mechanism of action was elucidated in this study. The obtained findings demonstrated that subacute vortioxetine treatment (5 and 10 mg/kg for 2 weeks) increased the reduced paw-withdrawal thresholds of diabetic rats both in the Randall–Selitto and Dynamic plantar tests. Moreover, the falling latencies of animals did not change in the Rota-rod assessments. These results suggest that vortioxetine administration significantly improved diabetes-induced hyperalgesia and allodynia responses in the rats without affecting their motor coordination. The vortioxetine (5 mg/kg)-induced antihyperalgesic and antiallodynic effects were reversed by AMPT, yohimbine, ICI 118,551, sulpiride and atropine pre-treatments, suggesting the involvement of the catecholaminergic system, α_2_- and β_2_-adrenoceptors, D_2/3_ dopaminergic receptors and cholinergic muscarinic receptors in the exhibited pharmacological activity, respectively. Moreover, the data from the immunohistochemical studies indicated that the inhibition of c-Fos overexpression in dorsal horn neurons also mediates the beneficial effect of this drug. Vortioxetine induced no difference in plasma glucose levels in diabetic rats. If clinical studies confirm these findings, the concomitant beneficial effect of vortioxetine on mood disorders and its neutral activity profile on glycemic control may make it an alternative drug for the treatment of neuropathic pain.

## 1. Introduction

Vortioxetine is an atypical antidepressant drug approved by the Food and Drug Administration and the European Medicines Agency in 2013. It has been marketed under the names Brintellix^®^, Trintellix^®^ and Fonksera^®^ to date [1,2]. This drug ((1-[2-(2,4-dimethylphenyl-sulfanyl)-phenyl]-piperazine) has multimodal actions as a modulator of serotonergic transmission. It acts as an inhibitor on the serotonin transporter, and also as an agonist (5-HT_1A_), partial agonist (5-HT_1B_) and antagonist (5-HT_1D_, 5-HT_3_, 5-HT_7_) of serotonin receptor subtypes [3,4]. Vortioxetine exerts its pharmacological effects in the central nervous system by regulating the release of various neurotransmitters such as noradrenaline, dopamine, histamine, acetylcholine, glutamate, gamma-aminobutyric acid, as well as serotonin [5,6,7,8,9,10].

Considering that the pharmacological action of vortioxetine is mediated by multiple neurotransmitter systems, it can be thought that this drug may have various therapeutic effects on the central nervous system. For example, a number of studies in the literature suggest that this drug may also be effective for treating both acute and chronic pain [7,11,12,13,14]. The beneficial effects of this drug have been shown in some neuropathic pain models, including reserpine-induced fibromyalgia [15], chronic constriction injury [16], chronic neuropathic orofacial pain [7,17] and oxaliplatin-induced neuropathy [18]. On the other hand, the efficacy of vortioxetine against diabetes-induced neuropathic pain, which includes serious metabolic, immune, neurotrophic and inflammatory components, has not been investigated so far [19,20,21].

Vortioxetine is revealed to act on the central nervous system mostly through serotonergic signaling [5,7]. On the other hand, it is known that selective serotonin reuptake inhibitor drugs (SSRIs), which show their effects only through the serotonergic system, are not very effective in the treatment of neuropathic pain, but the cholinergic and, especially, the catecholaminergic systems play critical roles in the treatment of neuropathy-related hyperalgesia and allodynia [22]. Studies to date have demonstrated that both of these endogenous systems also contribute to the various pharmacological activities of vortioxetine [12,17,18,23,24]. Therefore, in this study, we aimed to investigate the potential antihyperalgesic and antiallodynic activities of vortioxetine in the diabetes-induced neuropathic pain model and the possible involvement of the catecholaminergic and cholinergic systems in these effects. Furthermore, potential changes in the neuronal activity were also assessed in the spinal dorsal horn of the diabetic rats in the scope of this study.

## 2. Materials and Methods

### 2.1. Chemicals and Drugs

Streptozotocin (STZ), α-methyl-para-tyrosine methyl ester (AMPT), yohimbine, sulpiride, pregabalin and atropine were purchased from Sigma-Aldrich (St. Louis, MO, USA). ICI-118,551 was acquired from Cayman Chemical Company (Ann Arbor, MI, USA). Serum physiologic solution was provided by Osel (Beykoz, İstanbul, Turkey). Trisodium citrate and citric acid were from Merck (Darmstadt, Germany). Fonksera^®^ (Lundbeck, Copenhagen, Denmark) and Glifor^®^ (Bilim İlaç, Kocaeli, Turkey) were commercially provided for vortioxetine and metformin, respectively.

### 2.2. Animals

Inbred Sprague-Dawley rats (250–300 g weighted, male) were provided by the Research Unit for Experimental Animals of Anadolu University, Eskişehir, Turkey. The animals were maintained under stable conditions in well-ventilated rooms with a temperature set at 24 ± 1 °C and a 12 h/12 h dark-and-light cycle (lights on between 8.00 a.m. and 8.00 p.m.). Regular pellet feeds were provided for rats, and no restriction was applied to water/food. The experimental design of this study was approved by the Anadolu University Local Ethics Committee on Animal Experiments (ethical approval number 2020–33 and approval date 14 July 2020).

### 2.3. Induction of Diabetes Model on Animals 

For the induction of the diabetes model STZ, a glucose analogue with pancreatic toxicity was used [25]. It was prepared in a citrate buffer with a pH of 4.5 [26] and injected into the tail veins of rats at a dose of 50 mg/kg, following an overnight fast. The rats in the control group received an equivalent volume of citrate buffer. After the STZ injection, water bottles with 5 mmol/L glucose solution were put into the animal cages to minimize the risk of hypoglycemic shock. Glucose levels were measured using the Accu-Chek^®^ Performa Nano apparatus (Roche, Basel, Switzerland) from blood samples taken 72 h later. Rats having plasma glucose level greater than 300 mg/dL were defined as diabetic [26,27]. After initiating the experimental diabetes model, rats were kept for 4 weeks to allow for the development of neuropathic pain [27,28].

### 2.4. Pharmacological Treatment Protocol

Vortioxetine was administered at doses of 5 and 10 mg/kg (p.o.) to the diabetic animals for 14 days [29,30]. Control groups of healthy and diabetic animals received physiological saline solution, which was used in the dissolution of vortioxetine. Pregabalin (10 mg/kg, p.o.) and metformin (1 g/kg, p.o.) were used as positive controls for neuropathic pain and blood glucose experiments, respectively [26].

### 2.5. Measurement of Plasma Glucose Levels

After completion of the in vivo experiments, animals were fasted overnight, and blood glucose levels were measured 60 min after the last dose of vortioxetine on the 15th day.

### 2.6. Motor Coordination Experiments

Potential alterations in the motor coordination parameters of the animals were assessed by Rota-rod apparatus (device code 47700, Ugo Basile, Varese, Italy). The animals were trained for three days before the experiments. On the test day, the device was adjusted to a constant speed of 8 rpm, and the falling latencies of the rats over the rotating mill were recorded [31].

### 2.7. Neuropathic Pain Experiments

#### 2.7.1. Randall–Selitto Test

For the assessment of mechanical hyperalgesia, the Randall–Selitto apparatus was used (device code 37215, Ugo Basile, Varese, Italy). In this test, the dorsal regions of the rats’ hind paws were subjected to gradually rising pressure. The force (given in grams) that triggered paw withdrawal was assumed to be the mechanical nociceptive threshold. To protect the paws from any damage, the applied maximum force did not exceed the limit of 250 g [27,32].

#### 2.7.2. Dynamic Plantar Test

For the assessment of mechanical allodynia, a Dynamic plantar aesthesiometer instrument (device code 37450, Ugo Basile, Varese, Italy) was used. The animals were placed in transparent chambers (17 cm × 69 cm × 14 cm) which were on an elevated wire-mesh platform with a moveable component underneath applying escalating mechanical force with a steel rod. For the adaptation to the experimental environment, the animals were freely kept for 30 min in these plexiglass chambers before the tests. Subsequently, the device applied increasing force (2.5 g/s) to the plantar regions of the hind paw of the rats with the metal rod. The mechanical stimulation was increased spontaneously until the rats withdrew their paws. The system measured the force with an accuracy of 0.1 g [32]. In 5 min intervals, paw-withdrawal thresholds were recorded three times, and average values were calculated for each rat. A mechanical stimulus higher than 50 g was not administered to prevent paw injury [12,33].

### 2.8. Studies for the Underlying Mechanisms

Probable mechanisms underlying the antihyperalgesic and antiallodynic effects of vortioxetine were investigated with further studies. Possible involvement of catecholaminergic system in the activity was evaluated by using AMPT. This catecholamine synthesis inhibitor was injected intraperitoneally (i.p.) twice (24 and 1 h before the last vortioxetine administration) at a dose of 200 mg/kg [34]. Furthermore, possible involvements of α-adrenergic, β-adrenergic, dopaminergic and cholinergic receptors were investigated by using yohimbine (2 mg/kg, i.p., an α_2_-adrenoceptor blocker) [35,36], ICI 118,551 (1 mg/kg, i.p., a β_2_ -adrenoceptor blocker) [37], sulpiride (30 mg/kg, i.p., a dopamine D_2_/D_3_ receptor blocker) [38] and atropine (5 mg/kg, i.p., a non-selective muscarinic receptor blocker) [39], respectively. These agents were administered 15 min before vortioxetine administrations.

Doses and administration routes of the agents used in the mechanistic studies were selected by considering previous studies in our laboratory and the methods used in similar studies in the literature. Moreover, the mechanistic studies were performed with a low dose of vortioxetine (5 mg/kg), as there was no significant difference in the effects caused by the two doses tested.

**Figure 1:**
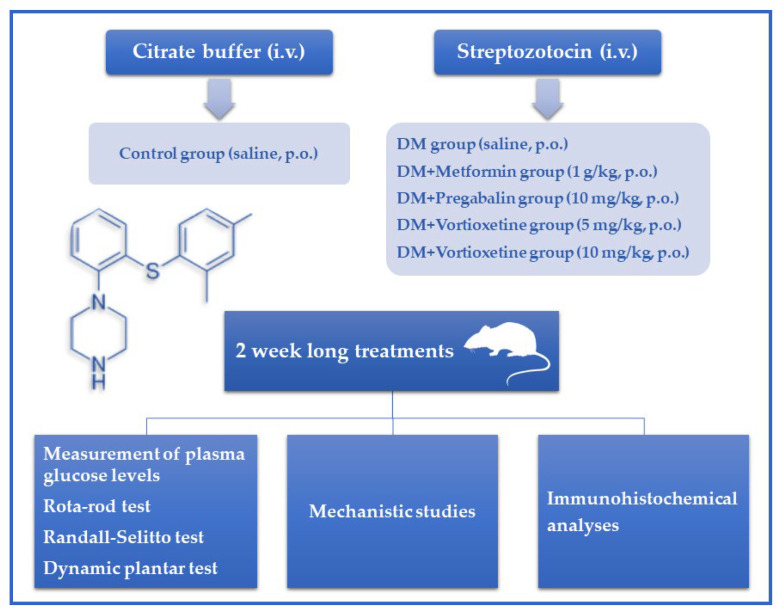
Summary of the experimental design.

Details of the experimental protocol are presented in Figure 1.

### 2.9. Immunohistochemical Analyses

Immunohistochemical analyses were performed after the completion of neuropathic pain experiments. Rats were perfused with 0.1 M phosphate buffered saline (PBS) and paraformaldehyde in PBS (4%, pH 7.4) following anesthesia induced by halothane, and then their L4-L5 spinal cord segments were dissected.

#### 2.9.1. Histopathological Procedure

Tissue samples were fixed overnight (at 4 °C) in 10% neutral buffer formaldehyde solution for light microscopy examination. Following the fixation process, tissue samples were placed in cassettes and washed under running water for 2 h. To remove water, the spinal tissues were passed through a series of increasing concentrations of alcohol (60%, 70%, 80%, 90%, 96% and 100%). The tissues were then subjected to xylol and embedded in paraffin.

#### 2.9.2. Immunohistochemical Staining

Transverse sections of 4 μm thickness were collected from the paraffin blocks and mounted on slides. Following the deparaffinization process, slides were passed through a decreasing alcohol series and rehydrated. A 1/10 diluted EDTA Buffer (pH = 8) (AP-9004-999 Thermo Scientific, Waltham, MA, USA) was applied to overcome antigen masking. Subsequently, in order to block endogenous peroxidase activity (for non-specific background staining), 3% H_2_O_2_ solution (TA-125-HP ThermoScientific, Waltham, MA, USA) was applied. Then the tissues were treated with PBS, which was followed by protein block solution (TA-125-PBQ Thermo Scientific, Waltham, MA, USA).

The spinal sections were incubated with 1:75 rabbit anti-*c-fos* antibody (GTX27963, Genetex, CA, USA) for 2 h. Then, Amplifier Quanto (TL-125-QPB) and, after that, HRP Polymer Quanto (TL-125-QPH) were applied for 30 min., rinsing in PBS at every step. Then, the sections were treated with DAB Chromogen (TA-125-HA Thermo Scientific, Waltham, MA, USA), washed and dehydrated. They were exposed to xylol and coverslipped with entellan.

#### 2.9.3. Microscopy and Immunohistochemistry

An Olympus CX31RTSF optical microscope (Olympus GmbH, Hamburg, Germany) with LCmicro version 2.1 imaging software (Olympus GmbH, Hamburg, Germany) and an integrated camera with a 4× objective lens was used to acquire photomicrographs. All sections were digitally captured using a 40× lens before being analyzed using the image processing and analysis tool ImageJ version 1.50i (U.S. National Institutes of Health, Bethesda, MD, USA). The c-Fos-positive cells corresponding to the regions of laminae I and II, the superficial layer of the medulla spinalis, were counted, and the mean neuronal count of the three sections from the L4-L5 lumbar segment of the spinal cord was determined for each animal [40].

### 2.10. Statistical Evaluation

The software package program GraphPad Prism version 8.4.3. (GraphPad Software, San Diego, CA, USA) was used for the statistical analysis. The data obtained from Randall–Selitto, Dynamic Plantar, Rota-rod tests, plasma glucose measurements and immunohistochemical analyses were evaluated by one-way analysis of variance (ANOVA) followed by Tukey’s HSD test for multiple comparisons. Data acquired from the mechanistic studies were analyzed by two-way ANOVA followed by the Bonferroni multiple comparison test. All values were given as the mean ± standard error of the mean (S.E.M.). Probability (*p*) values under 0.05 were considered significant.

## 3. Results

### 3.1. Effects of Vortioxetine Treatment on Blood Glucose Levels in Diabetic Rats

**Figure 2:**
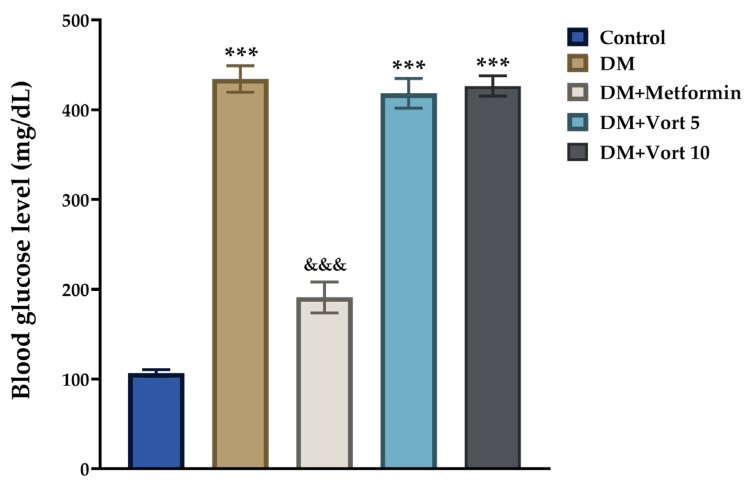
Blood glucose levels in normoglycemic rats administered with saline solution (control) and diabetic rats administered with saline (DM), 1000 mg/kg metformin (DM + Metformin), 5 mg/kg vortioxetine (DM + Vort 5) or 10 mg/kg vortioxetine (DM + Vort 10). Values are given as mean ± S.E.M. Significant difference against control group *** *p* < 0.001; significant difference against DM group ^&&&^ *p* < 0.001. One-way ANOVA, post hoc Tukey’s HSD multiple comparison test, *n* = 8.

In Figure 2, the vortioxetine (5 and 10 mg/kg/day) and metformin (1000 mg/kg) administration-induced alterations on blood glucose levels in diabetic rats are shown [F (4, 35) = 129.5, *p* < 0.001]. The multiple comparison tests revealed that vortioxetine treatment at both doses did not alter (*p* > 0.05) the hyperglycemia levels of diabetic rats.

### 3.2. Effects of Vortioxetine Treatment on Motor Coordination of Diabetic Rats 

**Figure 3:**
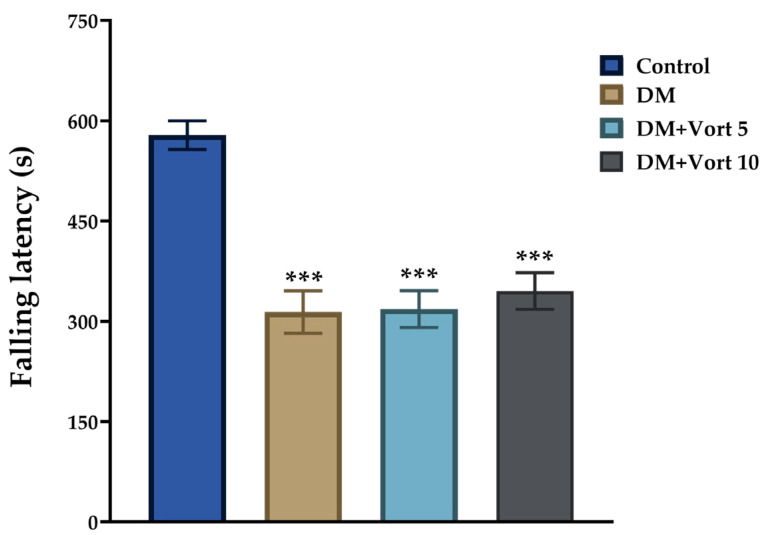
Falling latencies of normoglycemic rats administered with saline solution (control) and diabetic rats administered with saline (DM), 5 mg/kg vortioxetine (DM + Vort 5) or 10 mg/kg vortioxetine (DM + Vort 10) in the Rota-rod tests. Values are given as mean ± S.E.M. Significant difference against control group *** *p* < 0.001. One-way ANOVA, post hoc Tukey’s HSD multiple comparison test, *n* = 8.

The alterations in the motor coordination of diabetic rats following the administration of vortioxetine are presented in Figure 3 [F (3, 28) = 21.83, *p* < 0.001]. The results of the multiple comparison tests showed that vortioxetine administration did not affect (*p* > 0.05) the motor performances of diabetic rats.

### 3.3. Effects of Vortioxetine on Diabetes-Induced Neuropathic Pain 

#### 3.3.1. Randall–Selitto Test Results

**Figure 4:**
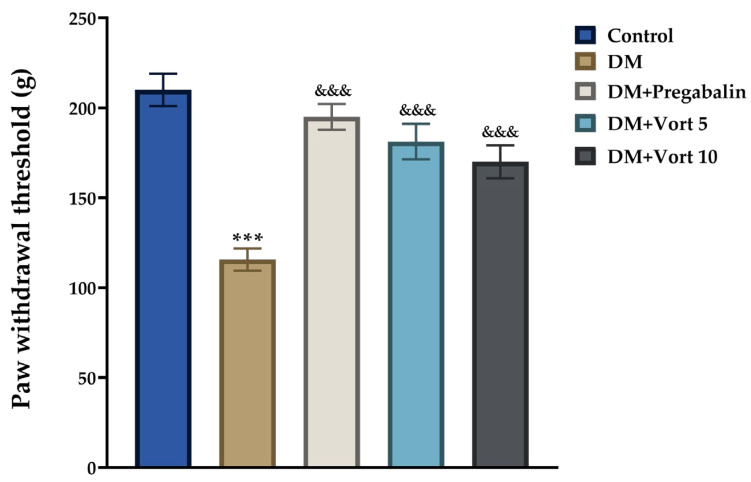
Paw withdrawal thresholds of normoglycemic rats administered with saline solution (control) and diabetic rats administered with saline (DM), 10 mg/kg pregabalin (DM + Pregabalin), 5 mg/kg vortioxetine (DM + Vort 5) or 10 mg/kg vortioxetine (DM + Vort 10) in the Randall–Selitto test. Values are given as mean ± S.E.M. Significant difference against control group *** *p* < 0.001; significant difference against DM group ^&&&^ *p* < 0.001. One-way ANOVA, post hoc Tukey’s HSD multiple comparison test, *n* = 8.

In Figure 4, the beneficial effects of vortioxetine (5 and 10 mg/kg/day) and pregabalin (10 mg/kg/day) treatments on mechanical nociceptive stimulus-induced hyperalgesia responses in diabetic rats are demonstrated [F (4, 35) = 18.61, *p* < 0.001]. The Tukey’s HSD multiple comparison test showed that both vortioxetine (*p* < 0.001) and pregabalin (*p* < 0.001) administration increased the reduced paw-withdrawal threshold values in diabetic animals.

#### 3.3.2. Dynamic Plantar Test Results

**Figure 5:**
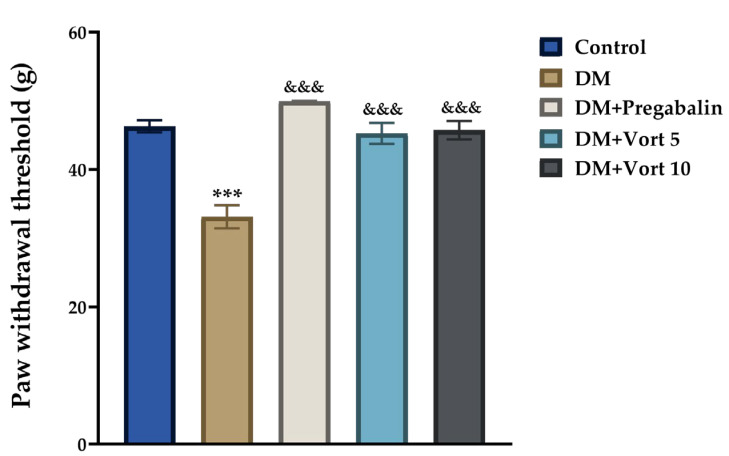
Paw withdrawal thresholds of normoglycemic rats administered with saline solution (control) and diabetic rats administered with saline (DM), 10 mg/kg pregabalin (DM + Pregabalin), 5 mg/kg vortioxetine (DM + Vort 5) or 10 mg/kg vortioxetine (DM + Vort 10) in the Dynamic plantar test. Values are given as mean ± S.E.M. Significant difference against control group *** *p* < 0.001; significant difference against DM group ^&&&^ *p* < 0.001. One-way ANOVA, post hoc Tukey’s HSD multiple comparison test, *n* = 8.

The data obtained from the Dynamic plantar tests after vortioxetine (5 and 10 mg/kg/day) and pregabalin (10 mg/kg/day) administration are presented in Figure 5 [F (4, 35) = 26.19, *p* < 0.001]. The results of the Tukey’s HSD multiple comparison tests revealed that the administration of vortioxetine at doses of 5 mg/kg (*p* < 0.001) and 10 mg/kg (*p* < 0.001) notably increased the decreased paw withdrawal thresholds of diabetic rats. Pregabalin also exhibited the expected antiallodynic efficacy following the 14-day treatments (*p* < 0.001).

### 3.4. Mechanistic Studies

#### 3.4.1. Participation of Catecholaminergic System in the Beneficial Effect of Vortioxetine on Diabetes-Induced Mechanical Hyperalgesia and Allodynia

**Figure 6:**
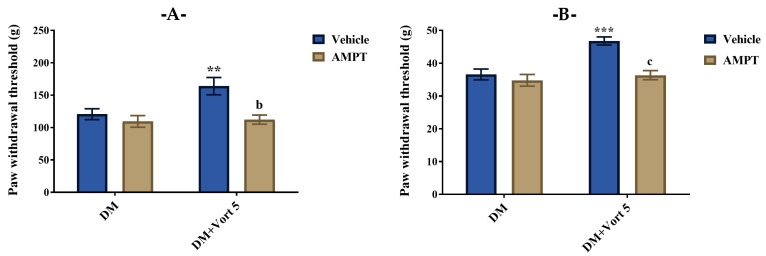
The effects of AMPT pre-treatment on antihyperalgesic (**A**) and antiallodynic (**B**) responses induced by the administration of 5 mg/kg vortioxetine in the Randall–Selitto and Dynamic plantar tests, respectively. Values are given as mean ± S.E.M. Significant difference against vehicle-administered diabetic group (DM) ** *p* < 0.01; *** *p* < 0.001; significant difference against vortioxetine-administered diabetic group (DM + Vort 5) ^b^ *p* < 0.01; ^c^ *p* < 0.001. Two-way ANOVA, post hoc Bonferroni multiple comparison test, *n* = 8.

The changes in the vortioxetine-induced antihyperalgesic and antiallodynic responses in the Randall–Sellito (6A) and Dynamic plantar (6B) tests following AMPT pre-treatment are presented in Figure 6.

In the Randall–Sellito test, a two-way ANOVA showed a significant treatment–AMPT administration interaction [F (1, 28) = 4.29, *p* < 0.05], as well as the significant main effects of treatment [F (1, 28) = 5.58, *p* < 0.05] and AMPT administrations [F (1, 28) = 10.34, *p* < 0.01]. Moreover, in the Dynamic plantar tests, a two-way ANOVA displayed a significant treatment–AMPT administration interaction [F (1, 28) = 7.99, *p* < 0.01] and the significant main effects of treatment [F (1, 28) = 14.74, *p* < 0.001] and AMPT administrations [F (1, 28) = 15.97, *p* < 0.001].

The post hoc analyses indicated that the AMPT pre-treatments significantly reversed the antihyperalgesic and antiallodynic responses in the Randall–Sellito (*p* < 0.01) and Dynamic plantar (*p* < 0.001) tests, respectively.

**Figure 7:**
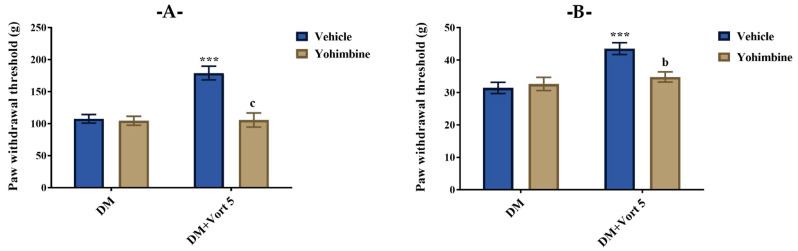
The effects of yohimbine pre-treatment on antihyperalgesic (**A**) and antiallodynic (**B**) responses induced by the administration of 5 mg/kg vortioxetine in the Randall-Selitto and Dynamic plantar tests, respectively. Values are given as mean ± S.E.M. Significant difference against vehicle-administered diabetic group (DM) *** *p* < 0.001; significant difference against vortioxetine-administered diabetic group (DM+Vort 5) ^b^ *p* < 0.01; ^c^ *p* < 0.001. Two-way ANOVA, post hoc Bonferroni multiple comparison test, *n* = 8.

In Figure 7, the alterations in the vortioxetine-induced antihyperalgesic responses in the Randall-Sellito (7A) and the antiallodynic responses in the Dynamic plantar (7B) tests following yohimbine pre-treatments are shown. A two-way ANOVA revealed the significant main effects of treatment [F (1, 28) = 16.11, *p* < 0.001], yohimbine administration [F (1, 28) = 17.89, *p* < 0.001] and the interaction between these factors [F (1, 28) = 15.18, *p* < 0.001] in the Randall-Sellito test. Furthermore, a two-way ANOVA analysis indicated the significant effects of treatment [F (1, 28) = 15.77, *p* < 0.001] and yohimbine administration factors [F (1, 28) = 4.42, *p* < 0.05] in the Dynamic plantar tests. There is also a significant interaction between the treatment and yohimbine administration factors [F (1, 28) = 7.65, *p* < 0.01].

The results of the Bonferroni multiple comparisons test showed that yohimbine pre-treatments significantly reversed the vortioxetine-induced antihyperalgesic responses in the Randall-Sellito (*p* < 0.001) experiments and the antiallodynic responses in the Dynamic plantar (*p* < 0.01) tests.

**Figure 8:**
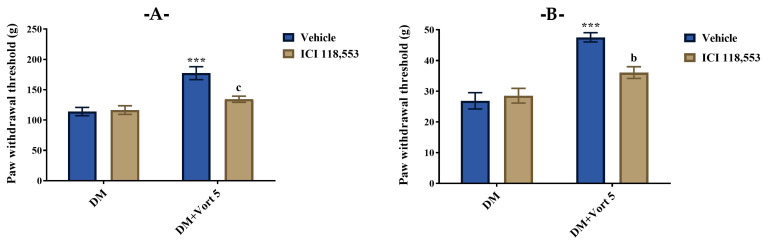
The effects of ICI 118,553 pre-treatment on antihyperalgesic (**A**) and antiallodynic (**B**) responses induced by the administration of 5 mg/kg vortioxetine in the Randall–Selitto and Dynamic plantar tests, respectively. Values are given as mean ± S.E.M. Significant difference against vehicle-administered diabetic group (DM) *** *p* < 0.001; significant difference against vortioxetine-administered diabetic group (DM+Vort 5) ^b^ *p* < 0.01; ^c^ *p* < 0.001. Two-way ANOVA, post hoc Bonferroni multiple comparison test, *n* = 8.

The effects of ICI 118,553 pre-treatments on the vortioxetine-induced antihyperalgesic and antiallodynic responses in the Randall-Sellito (8A) and Dynamic plantar (8B) tests are shown in Figure 8. A two-way ANOVA analysis displayed that both the treatment [F (1, 28) = 27.72, *p* < 0.001] and the antagonist factors [F (1, 28) = 6.89, *p* < 0.05] had an effect on the paw withdrawal thresholds of rats measured in the Randall-Sellito test. There was also a significant interaction between the treatment and antagonist factors [F (1, 28) = 8.69, *p* < 0.01]. In the Dynamic plantar tests, a two-way ANOVA revealed the significant effects of the treatment factor [F (1, 28) = 42.46, *p* < 0.001], ICI 118,553 administration factor [F (1, 28) = 5.15, *p* < 0.05] and the interaction between them [F (1, 28) = 9.23, *p* < 0.01].

The results of the Bonferroni multiple comparisons test showed that ICI 118,553 administration significantly reversed the vortioxetine-induced antihyperalgesic responses in the Randall-Sellito (*p* < 0.001) experiments and the antiallodynic responses in the Dynamic plantar (*p* < 0.01) tests.

**Figure 9:**
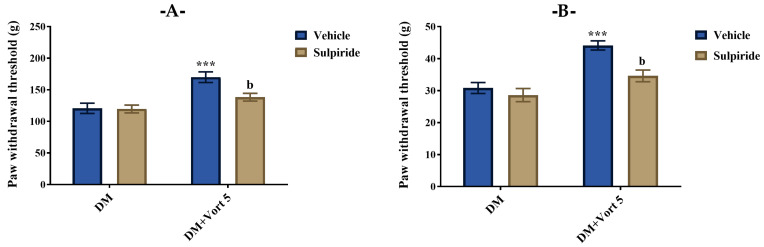
The effects of sulpiride pre-treatment on antihyperalgesic (**A**) and antiallodynic (**B**) responses induced by the administration of 5 mg/kg vortioxetine in the Randall–Selitto and Dynamic plantar tests, respectively. Values are given as mean ± S.E.M. Significant difference against vehicle-administered diabetic group (DM) *** *p* < 0.001; significant difference against vortioxetine-administrated diabetic group (DM+Vort 5) ^b^ *p* < 0.01. Two-way ANOVA, post hoc Bonferroni multiple comparison test, *n* = 8.

The effect of sulpiride pre-treatment on the antihyperalgesic effect of vortioxetine in the Randall-Sellito test (9A) and the antiallodynic effect in the Dynamic plantar (9B) test are shown in Figure 9. In the Randall-Sellito test, a two-way ANOVA indicated the significant effects of the treatment [F (1, 28) = 21.6, *p* < 0.001] and sulpiride administration [F (1, 28) = 5.06, *p* < 0.05] factors. This analysis also demonstrated a significant interaction between these two factors [F (1, 28) = 4.39, *p* < 0.05]. Similarly, in the Dynamic plantar tests, a two-way ANOVA analysis presented a significant interaction between the treatment and sulpiride administration factors [F (1, 28) = 4.30, *p* < 0.05]. There were also significant main effects of the treatment [F (1, 28) = 30.01, *p* < 0.001] and sulpiride administration [F (1, 28) = 11.10, *p* < 0.01] factors.

The multiple comparison tests revealed that sulpiride pre-treatments reversed the antihyperalgesic and antiallodynic effects of vortioxetine both in the Randall-Sellito (*p* < 0.01) and the Dynamic plantar (*p* < 0.01) tests, respectively.

#### 3.4.2. Participation of Cholinergic System in the Beneficial Effect of Vortioxetine on Diabetes-Induced Mechanical Hyperalgesia and Allodynia 

**Figure 10:**
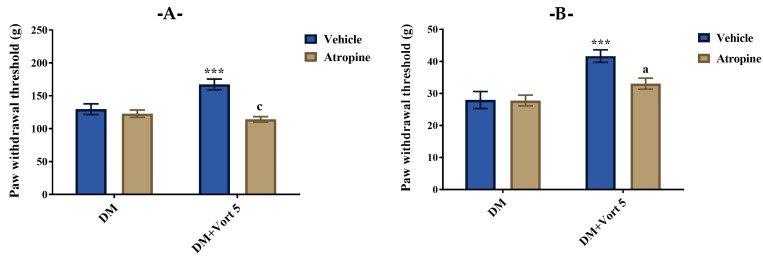
The effects of atropine pre-treatment on antihyperalgesic (**A**) and antiallodynic (**B**) responses induced by the administration of 5 mg/kg vortioxetine in the Randall–Selitto and Dynamic plantar tests, respectively. Values are given as mean ± S.E.M. Significant difference against vehicle-administered diabetic group (DM) *** *p* < 0.001; significant difference against vortioxetine-administered diabetic group (DM+Vort 5) ^a^ *p* < 0.05; ^c^ *p* < 0.001. Two-way ANOVA, post hoc Bonferroni multiple comparison test, *n* = 8.

In Figure 10, the effect of atropine pre-treatment on the antihyperalgesic and antiallodynic effects of vortioxetine in the Randall-Sellito (10A) and Dynamic plantar (10B) tests is presented. In the Randall-Sellito test, a two-way ANOVA demonstrated the significant effects of the treatment [F (1, 28) = 4.61, *p* < 0.05] and atropine administration factors [F (1, 28) = 19.91, *p* < 0.001] together with a significant interaction between them [F (1, 28) = 11.84, *p* < 0.01]. Furthermore, in the Dynamic plantar tests, a two-way ANOVA revealed the significant effects of the treatment factor [F (1, 28) = 21.60, *p* < 0.001], the atropine administration factor [F (1, 28) = 4.61, *p* < 0.05] and their interaction [F (1, 28) = 4.29, *p* < 0.05].

The post hoc analyses showed that the antihyperalgesic and antiallodynic effects of vortioxetine were significantly antagonized following atropine pre-treatments in the Randall-Sellito (*p* < 0.001) and the Dynamic plantar (*p* < 0.05) tests, respectively.

### 3.5. Vortioxetine-Induced c-Fos Immunoreactivity in the Dorsal Horn of Diabetic Rats

**Figure 11:**
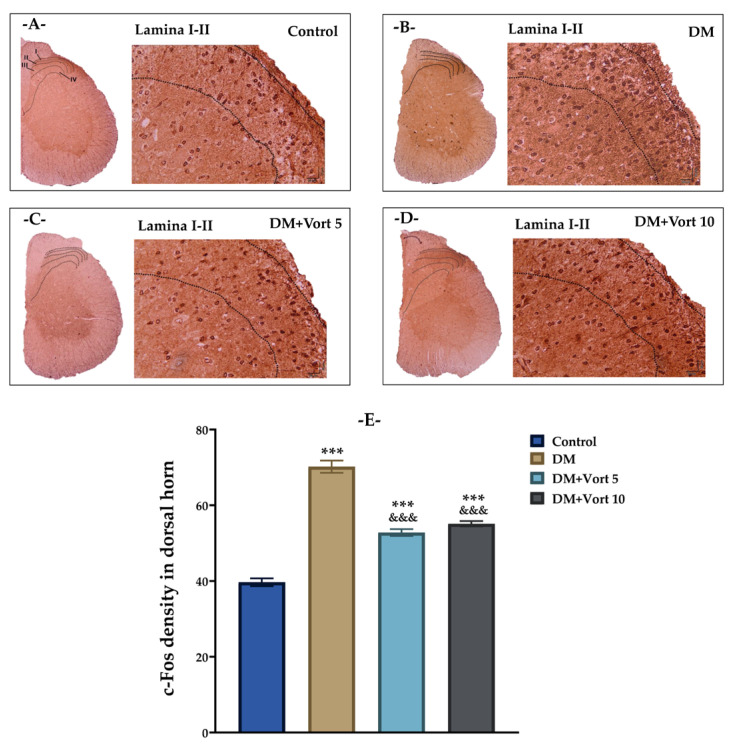
Representative images of c-Fos-immunoreactivities in the superficial layer (laminae I and II) of the lumbar spinal cord (L4–L5 segment) dorsal horn of control (**A**), diabetic (DM) (**B**), 5 mg/kg vortioxetine-administered diabetic (DM+Vort 5) (**C**) and 10 mg/kg vortioxetine-administered diabetic (DM+Vort 10) (**D**) rats. Scale bar = 20 μm. c-Fos densities in dorsal horn of control, diabetic (DM), 5 mg/kg vortioxetine-administered diabetic (DM+Vort 5) and 10 mg/kg vortioxetine-administered diabetic (DM+Vort 10) rats (**E**). Significant difference compared to the control group *** *p* < 0.001; significant difference compared to the DM group ^&&&^ *p* < 0.001; one-way ANOVA followed by Tukey’s HSD multiple comparison test, *n* = 8.

Representative images of c-Fos immunoreactivities in the dorsal horn of diabetic rats are presented in Figure 11.

It was observed that the induction of diabetes increased c-Fos immunoreactivities (Figure 11A,B), and this enhancement was reduced by 5 mg/ kg (Figure 11C) and 10 mg/kg (Figure 11D) vortioxetine administrations.

The numerical densities of c-Fos immunopositive cells in the dorsal horn are shown in Figure 11E. The Tukey’s HSD multiple comparisons test showed that diabetic rats had significantly higher densities of c-Fos immunoreactive cells than those of the control group (*p* < 0.001), and vortioxetine treatment at both doses decreased these enhanced densities of c-Fos immunopositive cells [F (3, 28) = 124.1; *p* < 0.001].

## 4. Discussion

In this study, based on previous reports presenting the therapeutic potential of vortioxetine for acute [12,13], inflammatory [11] and neuropathic pain [7,14], the potential efficacy of this drug against diabetes-induced hyperalgesia and allodynia responses was investigated in rats. Further mechanistic studies were conducted in order to elucidate the promising contributions of the catecholaminergic and cholinergic systems to the antihyperalgesic and antiallodynic activities of vortioxetine. Additionally, the vortioxetine-induced changes in the blood glucose levels of diabetic rats and the possible alterations in c-Fos expression in the dorsal horns of their spinal cords were also investigated.

We tested the potential activity of vortioxetine on hyperglycemia levels in diabetic animals in the first step of this study. The data obtained revealed that vortioxetine treatment had no beneficial effect on the hyperglycemia levels of diabetic rats. On the other hand, metformin administration significantly reduced the elevated blood glucose values, as expected (Figure 2). Since vortioxetine has no effect on hyperglycemia, it can be expected that this drug will not have any adverse effects on glycemic control in patients with diabetes.

It is known that the data obtained from neuropathic pain tests can be affected by possible changes in the motor performance of experimental animals. Therefore, in the second stage of this study, the possible effects of vortioxetine on the motor coordination of the rats in the experimental groups were investigated. The results obtained from the Rota-rod tests revealed that the falling latencies of diabetic rats were shortened compared to the normoglycemic group, suggesting that the motor coordination of these animals was impaired. These findings support the results of previous studies reporting impaired motor performance in diabetic animals [28,41,42]. On the other hand, vortioxetine administrations did not change the impaired motor coordination of diabetic rats (Figure 3). This finding is important as it revealed that the data from the neuropathic pain experiments in this study were not affected by any changes in the motor performance of the animals.

In the third step of our study, the effect of vortioxetine against diabetes-related neuropathic pain was investigated. The data from the Randall–Selitto tests showed that diabetic rats had lower paw withdrawal thresholds to mechanical nociceptive stimuli compared to normoglycemic mice. These data indicated that a mechanical hyperalgesic response developed in diabetic rats (Figure 4). Similarly, in the Dynamic plantar test, diabetic rats had lower paw withdrawal thresholds to non-nociceptive mechanical stimuli compared to normoglycemics, indicating that they developed mechanical allodynia (Figure 5). All these findings revealed that the diabetic neuropathic pain model was successfully induced in our study. Vortioxetine treatment, at 5 and 10 mg/kg for 2 weeks, increased the reduced paw withdrawal thresholds of the diabetic animals to the control levels in both tests (Figure 4 and Figure 5). However, there was no difference in antihyperalgesic/antiallodynic efficacy between the two doses of vortioxetine. Pregabalin, which was used as a positive control in the neuropathic pain experiments, also showed the expected antihyperalgesic and antiallodynic effects.

Parallel to our work, in a study evaluating the effect of vortioxetine on pain threshold in mice with reserpine-induced fibromyalgia-like symptoms, the administration of this drug at a dose of 10 mg/kg (i.p.) was shown to alleviate tactile allodynia [15]. In another study, Zuena et al. investigated the efficacy of vortioxetine against neuropathic pain in mice using a model of chronic constriction injury. The authors reported that the administration of this drug at a daily dose of 10 mg/kg (i.p.) for 27 days significantly increased the mechanical pain thresholds of the animals without changing their motor activity [16]. In a different study examining the effects of vortioxetine on pain hypersensitivity in a model of oxaliplatin-induced neuropathy in mice, it was suggested that both repeated prophylactic and acute therapeutic regimens of this drug (1–10 mg/kg, p.o.) dose-dependently reduce mechanical allodynia in the von Frey test and cold allodynia in the acetone test [18]. All these previous papers reporting the efficacy of vortioxetine on neuropathic pain support the presented preclinical findings of this study. Indeed, the efficacy of vortioxetine (10 mg, 15 mg and 20 mg) against neuropathic pain conditions was demonstrated in a clinical study on patients with burning mouth syndrome. The clinical findings of the aforementioned study support the hypothesis that vortioxetine may also have clinical therapeutic efficacy against diabetes-related neuropathic pain [17].

After the antihyperalgesic and antiallodynic effects of vortioxetine were revealed, the focus was on elucidating the pharmacological mechanisms mediating this effect. Vortioxetine is a serotonergic modulatory drug that has an agonistic effect on 5-HT_1A_, a partial agonistic effect on 5-HT_1B_ and antagonistic effects on the 5-HT_3_, 5-HT_7_ and 5-HT_1D_ receptor subtypes. It also has serotonin transporter inhibitory activity [3,4]. In the literature, the effectiveness of this drug on nociceptive and neuropathic pain has been associated with the serotonergic system [7,12]. However, it is known that pure SSRI drugs are insufficient to treat neuropathic pain [22,43,44]. Furthermore, they have been reported to be less effective than selective noradrenaline reuptake inhibitor (SNRI) drugs for the management of diabetic neuropathic pain [45,46]. Therefore, it is likely that the antihyperalgesic and antiallodynic effects of vortioxetine revealed in this study were also mediated by endogenous mechanisms other than the serotonergic system. Hence, we focused on noradrenaline, dopamine and acetylcholine, which are neurotransmitters known to be closely associated with neuropathic pain [43,47,48] and whose central levels are affected by vortioxetine administration [49,50].

The involvement of the noradrenergic and dopaminergic systems in the antihyperalgesic and antiallodynic activities of vortioxetine was tested using a selective tyrosine hydroxylase enzyme inhibitory agent, AMPT. The inhibition of this enzyme is known to reduce catecholamine synthesis. The administration of AMPT to rats as described in this study has been reported to cause a 50–60% decrease in noradrenaline levels in the central nervous system [51,52]. Our experimental data showed that AMPT pre-treatment completely reversed the vortioxetine-induced antihyperalgesic effect in the Randall-Selitto tests and the antiallodynic effects in the Dynamic plantar experiments (Figure 6). These results suggest that the beneficial effect of vortioxetine is at least partially related to the enhancement of catecholamines in the synaptic clefts. Parallel to these experimental results, vortioxetine treatment has been shown to increase noradrenaline levels in the locus coeruleus and dopamine levels in the prefrontal cortex [8]. In fact, these increases have been associated with the 5HT_3_ receptor antagonistic [9] and 5-HT_1A_ agonistic effects of this drug [10]. Indeed, in a study by Micov et al., the antiallodynic activity of vortioxetine in a model of oxaliplatin-induced neuropathy was associated with increased amounts of noradrenaline (along with serotonin) in the brainstem of mice [18]. These findings support the results of our AMPT studies.

After revealing that the antihyperalgesic and antiallodynic activities of vortioxetine are mediated by the catecholaminergic system, the potential involvement of the catecholaminergic receptors, which are known to be closely related to neuropathic pain and analgesia processes [53,54,55], was investigated in this study. To this end, antagonism studies were conducted with yohimbine, an α_2_-adrenergic receptor-blocking agent, ICI 118,551, a β_2_-adrenergic receptor blocker, and sulpiride, a D_2_/D_3_-dopaminergic receptor-blocking agent. Pre-treatments with these antagonists significantly reversed both the antihyperalgesic and antiallodynic activities of vortioxetine in diabetic rats (Figure 7, Figure 8 and Figure 9). The obtained findings indicate that the α_2_-adrenergic, β_2_-adrenergic and D_2_/D_3_-dopaminergic receptors play roles in the beneficial effects of vortioxetine against diabetes-induced neuropathic pain.

In addition to the catecholaminergic system, the cholinergic system and, particularly, the cholinergic muscarinic receptors are endogenous components that play a role in the modulation of pain and analgesia processes [56,57,58]. Hence, in this study, the possible contributions of muscarinic receptors to the antihyperalgesic and antiallodynic effects of vortioxetine were investigated using atropine, a non-selective muscarinic receptor antagonist agent. The data obtained indicated that atropine pre-treatment antagonized the effect of vortioxetine on diabetes-induced hyperalgesia and allodynia (Figure 10). These findings pointed out that muscarinic receptors, as well as the α_2_-, β_2_- and D_2_/D_3_-catecholaminergic receptors, participate in the aforementioned effect of vortioxetine. The results of a previous study by Todorović and co-workers suggesting that the analgesic activity of vortioxetine against trigeminal, visceral and somatic inflammatory pain is at least partially mediated by the α_2_/β_1_-adrenergic and muscarinic cholinergic receptors support the findings of our mechanistic studies [11].

c-Fos, which is the protein of the protooncogene *c-fos*, is involved in the signal transduction cascade that links extracellular events to intracellular adaptation. It is known that, although basal c-Fos expression is very low, high-threshold noxious stimuli can cause a very dramatic increase in c-Fos expression in spinal cord dorsal horn neurons [59]. Since c-Fos expression after noxious stimuli is specific, rapid and robust, it has been extensively used as a tool for the study of neural correlates of nociception and as a neuronal marker for examining the effectiveness of analgesic compounds [60,61,62]. Therefore, the potential effects of vortioxetine treatment on c-Fos immunoreactivity in the spinal horns of diabetic rats were investigated in this study. The findings obtained from the immunohistochemical studies revealed that c-Fos-positive cells in laminae I and II of the dorsal horns were increased in diabetic rats when compared to those in normoglycemic animals. These data support the results of previous studies showing c-Fos overexpression following peripheral and central nerve injury [63,64,65]. Moreover, some previous reports have also demonstrated the enhanced c-Fos levels in the spinal dorsal root of diabetic animals [40,66,67]. On the other hand, vortioxetine treatment both at 5 and 10 mg/kg doses significantly reduced these augmented c-Fos-positive cells in the L4-L5 dorsal horn neurons of diabetic rats (Figure 11). There was no difference between the 5 and 10 mg/kg doses of vortioxetine in terms of changes in c-Fos levels. Based on the higher activity of spinal dorsal horn neurons in diabetic animals [66,68,69] and the vortioxetine-induced decrease in the levels of c-Fos, a marker of neuronal activation, it can be suggested that vortioxetine inhibits the hyperexcitability of spinal cord dorsal horn neurons in diabetic rats and thus reduces diabetes-induced neuropathic pain.

Although in this study it was shown that the catecholaminergic and cholinergic systems are involved in the antihyperalgesic and antiallodynic effects of vortioxetine on diabetes-induced neuropathic pain, other possible mechanisms (such as glutaminergic, GABAergic, opioidergic, nitrergic, etc.) underlying these effects still remain to be resolved. In addition, it is clear that molecular studies on how vortioxetine treatment changes the levels and functions of other pain-related endogenous substances will contribute to elucidating the mechanism of action of vortioxetine.

In the study of Todorović et al., the efficacy of vortioxetine against inflammatory pain was demonstrated using trigeminal, visceral and somatic inflammatory pain models. In the mentioned study, vortioxetine was revealed to reduce the mice’s face rubbing times (5–20 mg/kg) in the second phase of the orofacial formalin test and the number of writhings in the acetic acid writhing test (10–20 mg/kg). On the other hand, this drug was also shown to reduce the paw pressure difference in a carrageenan-induced paw-inflammation model (1–10 mg/kg) in rats [11]. Since the pathophysiology of diabetic neuropathy is closely related to inflammation, it might also be possible to associate the antihyperalgesic and antiallodynic activities of vortioxetine against diabetic neuropathic pain with the activity of this drug against inflammatory pain. This issue needs to be elucidated with further studies.

## 5. Conclusions

To the best of our knowledge, this is the first study demonstrating the antihyperalgesic and antiallodynic activities of vortioxetine against diabetic neuropathic pain. The obtained data suggest that the α_2_-, β_2_- and D_2_/D_3_-catacholaminergic and muscarinic receptors together with the inhibition of c-Fos overexpression mediate the beneficial effect of vortioxetine.

In line with these findings, it is clear that there is a need for clinical studies investigating the efficacy of vortioxetine in diabetic patients with neuropathic pain. In addition to its antidepressant effect, the fact that vortioxetine does not affect glycemia levels in rats may make it possible for this drug to have extra therapeutic advantages in diabetic patients. This recommendation should be tested in well-designed clinical studies comparing the efficacy of vortioxetine with other analgesic drugs.

## Data Availability

All relevant data are included within the manuscript. The raw data are available on request from the corresponding author.
